# 

**Published:** 1863-06

**Authors:** 


### CHICAGO MEDICAL JOURNAL.

Terms, $2.00 per Annum.

### CONTENTS OF THE JUNE NUMBER.

PAGE.

ORIGINAL COMMUNICATIONS.

Report of Surgical Cases treated at the Battles of Pea Ridge and Prairie Grove, Ark. Primary and Secondary Amputations, Resections, Exsections, etc., etc. By E. A. Clark, M. D., Surgeon 8th Missouri Volunteer Cavalry .......... 241

Calomel in the Army .......... 254

SELECTED.

Cases Illustrative of the so-called "Congestive Fever," or Cerebro-Spinal Affection. By J. Baxter Upham, M. D., Surgeon in charge of Stanley Gen. Hospital, Eighteenth Army Corps.......... 260

Strychnia as a Poison. By Thos. D. Mitchell, M. D., Prof. of Materia-Medica and Therapeutics, in Jefferson Medical College, Phila.......... 271

Meeting of the "American" Medical Association.......... 276

EDITORIAL AND MISCELLANEOUS .......... 285

